# Vanadyl sulfate-enhanced oncolytic virus immunotherapy mediates the antitumor immune response by upregulating the secretion of pro-inflammatory cytokines and chemokines

**DOI:** 10.3389/fimmu.2022.1032356

**Published:** 2022-11-28

**Authors:** Nouf Alluqmani, Anna Jirovec, Zaid Taha, Oliver Varette, Andrew Chen, Daniel Serrano, Glib Maznyi, Sarwat Khan, Nicole E. Forbes, Rozanne Arulanandam, Rebecca C. Auer, Jean-Simon Diallo

**Affiliations:** ^1^ Centre for Innovative Cancer Research, Ottawa Hospital Research Institute, Ottawa, ON, Canada; ^2^ Department of Biochemistry, Microbiology and Immunology, Faculty of Medicine, University of Ottawa, ON, Canada; ^3^ Research Center, Molecular Oncology Department King Faisal Specialist Hospital and Research Center, Riyadh, Saudi Arabia; ^4^ Department of Surgery, University of Ottawa, Ottawa, ON, Canada

**Keywords:** combined immunotherapy, VSVΔ51, vanadyl sulfate, antitumor immunity, IL-12, IL-6, infected cell vaccine, interferon-gamma

## Abstract

Oncolytic viruses (OVs) are promising anticancer treatments that specifically replicate in and kill cancer cells and have profound immunostimulatory effects. We previously reported the potential of vanadium-based compounds such as vanadyl sulfate (VS) as immunostimulatory enhancers of OV immunotherapy. These compounds, in conjunction with RNA-based OVs such as oncolytic vesicular stomatitis virus (VSVΔ51), improve viral spread and oncolysis, leading to long-term antitumor immunity and prolonged survival in resistant tumor models. This effect is associated with a virus-induced antiviral type I IFN response shifting towards a type II IFN response in the presence of vanadium. Here, we investigated the systemic impact of VS+VSVΔ51 combination therapy to understand the immunological mechanism of action leading to improved antitumor responses. VS+VSVΔ51 combination therapy significantly increased the levels of IFN-γ and IL-6, and improved tumor antigen-specific T-cell responses. Supported by immunological profiling and as a proof of concept for the design of more effective therapeutic regimens, we found that local delivery of IL-12 using VSVΔ51 in combination with VS further improved therapeutic outcomes in a syngeneic CT26WT colon cancer model.

## 1 Introduction

Oncolytic viruses (OVs) are versatile and tumor-selective anticancer treatment platforms. OVs are selected and/or engineered viruses that preferentially infect, replicate in, and kill cancer cells (1–3). OVs also have immunostimulatory effects that promote antitumor immune responses. They successfully interrupt the established tumor microenvironment to overcome the resistance to immunological attack and the inhibition of immune lymphoid functions (4, 5).

The field of OV therapy has reached a new level of maturity with the historical approval of Imlygic^®^ for the treatment of advanced melanoma (6) and the more recent approval of Delytact (G47Δ; teserpaturev), a triple-mutated, replication-conditional herpes simplex virus type 1 (HSV-1) for glioblastoma (7). However, Imlygic has not achieved the anticipated level of commercial success, and most recently did not show significant clinical benefit in combination with immune checkpoint inhibitors (ICI) in phase III trials.

Beyond HSV-1, there are several promising OV platforms and opportunities for improvement of therapeutic efficacy. Among others, the efficacy of OV therapy has been shown to depend heavily on the successful delivery and spread of these agents within malignancies to redirect the immune response toward the tumor (5). Our group has discovered various small molecule enhancers of OVs that can be integrated in therapeutic regimens to potentiate the effects of OV immunotherapies. These molecules suppress and/or modulate the antiviral response thereby improving the efficacy of OVs, especially in OV-resistant tumors (8, 9). We recently uncovered the potential of vanadate and other vanadium-based compounds such as vanadyl sulfate (VS) as a unique category of immunostimulatory OV enhancers, showing high-potential for combination with RNA-based oncolytic viruses, such as the oncolytic vesicular stomatitis virus (VSVΔ51) as well as Newcastle disease virus (NDV) (10, 11).

Vanadium is a transition metal that has been shown to block protein phosphatase activity and disrupt the balance between cellular protein kinases and protein phosphatases. Vanadium compounds have diverse pharmacological activities and have been explored as insulin-mimetics in human trials owing to their capacity to inhibit protein tyrosine phosphatase 1B (PTP1B) and thereby activate the insulin receptor (12, 13). While vanadium compounds were shown to be safe and are currently used as body-building dietary supplements, they have not yet shown sufficient efficacy to be approved by regulators as treatments for diabetes. More recently, vanadium compounds have been tested as anticancer agents in a variety of cancer cell lines and animal models due to their effects as potential cancer therapeutics (14–18).

Previous studies from our group showed that sodium orthovanadate (vanadate) alters kinase/phosphatase homeostasis in VSVΔ51-infected cancer cells, leading to a shift from the traditional virus-induced antiviral type I interferon (IFN) response towards a type II IFN response (10). This shift, associated with the simultaneous downregulation of STAT2 (signal transducer and activator of transcription) and parallel activation of STAT1, results in improved virus growth and the up-regulation of many type-II IFN-regulated pro-inflammatory cytokines and leukocyte chemoattractants (e.g. CXCL9, CXCL10), a phenomenon observed in both human 786-0 and mouse CT26WT cell lines (9, 10). Recent studies have further shown that the observed effect on STAT1/STAT2 ratios involves vanadate/VSVΔ51 induced hyperactivation of the the epidermal growth factor receptor (EGFR) receptor and downstream pathways including NF-kB (Nuclear Factor Kappa B) (19). Supporting this mechanism, Gefitinib, an EGFR antagonist, abrogated the enhancing effect of vanadium *in vivo* in a syngeneic CT26WT model (19).

In several immunocompetent tumor models including but not limited to CT26WT, DBT, and 4T1, combined vanadate/VSVΔ51 improved viral spread within the tumor, delaying tumor progression and leading to both long-term remission and persistent antitumor immunity in cured animals (10). Notably, approximately 80% of DBT- (glioma) tumor-bearing mice and 20% of CT26WT (colon) tumor-bearing mice demonstrated complete remission after the combination treatment. Additionally, vanadate/VSVΔ51 combination treatment led to an enhancement in T cell recruitment to tumor sites with significantly increased interferon-gamma (IFN-γ) producing CD8+ T cells, particularly in treatment-responsive animals (10). A similarly profound therapeutic benefit was observed in another study using NDV in combination with vanadyl sulfate (VS), although in this context innate immune mechanisms predominate (11). Altogether, a better understanding of the *in vivo* immunological mechanisms involved in eliciting the improved therapeutic benefit provided by vanadium compounds is critical to inform the design of improved OV therapeutic regimens. In this study we sought to better understand the *in vivo* immunological mechanisms that lead to improved antitumor responses brought about from the combination of VS and VSVΔ51 in order to devise strategies to further drive therapeutic benefit.

## 2 Results

### 2.1 VS+VSVΔ51 combination treatment upregulates the secretion of pro-inflammatory chemokines and cytokines *in vivo*

**Figure 1:**
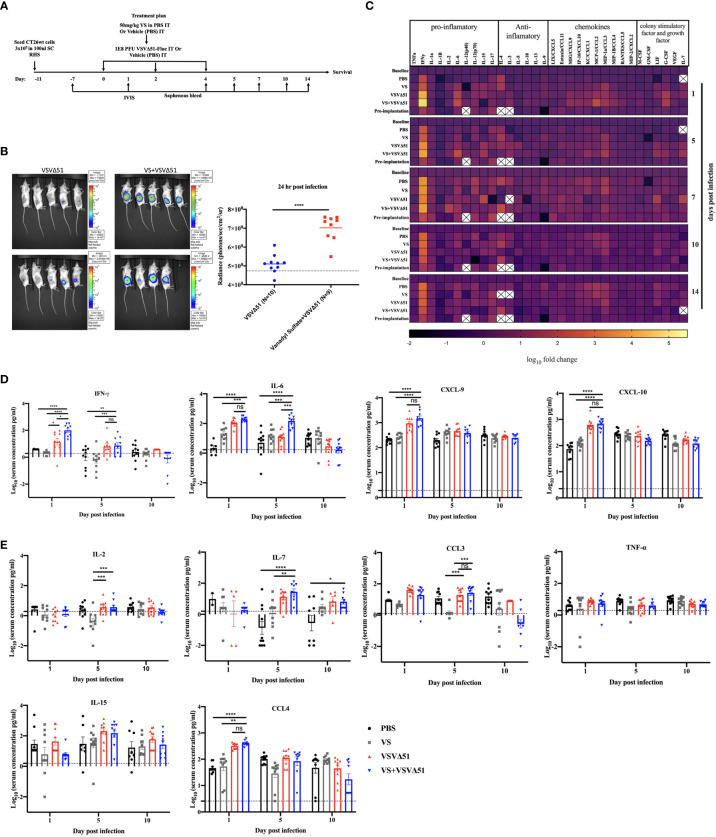
VS+VSVΔ51 co-treatment upregulates the secretion of pro-inflammatory chemokines and cytokines 1 and 5 days post the treatment. **(A)** Schematic representation of the treatment schedule: CT26WT-tumor bearing mice received a total of three doses delivered intratumorally of vanadyl sulfate (50mg/kg) and VSVΔ51-fluc (1E8 PFU) or monotreatment injections with PBS, vanadyl sulfate (50mg/kg), or VSVΔ51-fluc (1E8 PFU) alone over 5 days. **(B)** Twenty-four hours post the first treatment, viral replication was monitored by IVIS. Representative bioluminescence images of mice. Scale represented in photons, bar indicate mean. P=0.0007 as compared to mock-treated condition. **(C)** Heatmap represented the log_10_ fold change per condition of pooled sera for all the treated time points, normalized to day 7 post implantation as baseline. **(D, E)** The concentration of Cytokines and chemokines pg/ml for each group for each individual mouse, data transformed into log_10_. (n=10 per condition), line indicates the detection concentration, Mean ± SEM; * P=0.01, ** P=0.003, *** P=0.001,****P<0.0001, Two-way ANOVA.

To explore the immunological mechanisms that lead to improved antitumor responses brought about from the combination of VS+VSVΔ51, we chose to focus on the CT26WT colon cancer model (syngeneic in Balb/C). Subcutaneously implanted CT26WT tumors are inherently resistant to VSVΔ51 and while monotherapy is not curative, improved survival can be observed using a vanadate/VSVΔ51 therapeutic combination in this model, leading to approximately 20% complete remissions (10). Improved virus spread, as measured by VSVΔ51-encoded luciferase transgene-associated luminescence using an *in vivo* imaging system (IVIS), is also observed in this model (10). To confirm that similar effects were achievable using the body building supplement VS, mice were implanted with the CT26WT cells, and when tumors reached a volume of 100 mm^3^, three doses delivered intratumorally of VS (50 mg/kg) or PBS, followed by 1E8 plaque forming units (PFU) VSVΔ51 (encoding luciferase) or PBS were administrated over the course of 5 days (**Figure 1A**). In line with previous studies with vanadate, improved viral replication elicited by VS was confirmed by IVIS imaging 1 day post the first treatment (**Figure 1B**). The combination treatment was well-tolerated but accompanied with a limited and transient decrease in body weight followed by quick recovery within the following week (**Figure S1**).

A multiplex assay was subsequently performed using collected sera to evaluate the secretion of a panel of pro-inflammatory chemokines and cytokines in the blood at various time points following the treatment (**Figures 1C, D, E**). Pooled sera were used to map general trends in cytokine profiles over time (**Figure 1C**). Further analyses using sera from individual mice were carried out to confirm trends.

Our findings from these analyses revealed that, apart from increasing Interleukin-6 (IL-6) on day 1 (**Figure 1D**), VS had little impact on overall cytokine secretion. On the other hand, VSVΔ51 alone significantly increased the secretion of proinflammatory cytokines and chemokines including IFN-γ, CXCL9, CXCL10, CCL3, CCL4, and IL15 compared with VS and PBS groups (**Figures 1D, E**). Importantly, we observed significant increases in the secretion of a subset of cytokines and chemokines in the group that received the combined therapy as compared to monotherapies. Most notably, we measured a 5-fold increase in IFN-γ secretion 1-day post-infection ((**Figures 1C, D**), p-value=0.0001 compared to PBS and VS, and p-value=0.01 compared to VSVΔ51 alone, **Figure S2**). IL-6, an IFN-γ responsive cytokine, increased most significantly at 5 days post treatment (**Figures 1C, D**). In addition to IL-6, IFN-γ is known to upregulate the secretion of pro-inflammatory cytokines and chemokines. This includes CXCL-9 and CXCL10, which we have previously shown to be upregulated through vanadate/VSVΔ51 combination treatment in CT26WT cells *in vitro* (10). However, while average levels were highest coinciding with the peak of IFN-γ secretion, there was no statistically significant difference in CXCL9 when comparing VSVΔ51 to VSVΔ51 in combination with VS (p=0.1, **Figure S2**).

### 2.2 Combined VS+VSVΔ51 therapy enhances antigen-specific antitumor immune response

**Figure 2:**
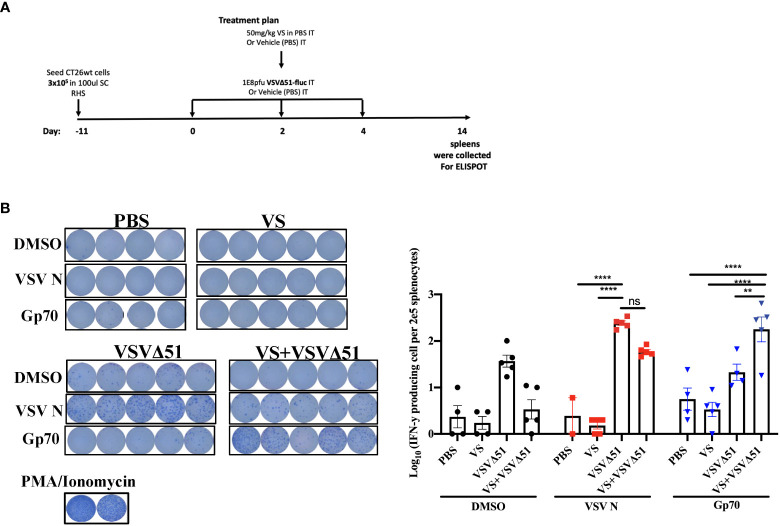
VS plus VSVΔ51 combination therapy further induces antigen specific immune response 14 days post treatment. **(A)** Schematic representation of the treatment schedule: after 12 days, CT26WT-tumor bearing mice received a total of three doses delivered intratumorally of vanadyl sulfate (50mg/kg) and VSVΔ51-fluc (1E8 PFU) or monotreatment injections with PBS, vanadyl sulfate (50mg/kg), or VSVΔ51-fluc (1E8 PFU) alone over 5 days. **(B)** Representative plates from an ELISPOT assay, splenocytes from treated mice were processed 14 days post treatment, then co-cultured with either DMSO, VSV N, or gp70 peptides for 20 hours, PMA and ionomycin were used as a positive control. Graph shows quantification of log_10_ of the number of IFN-γ producing cells per 2E5 splenocytes. N = 5 per condition, Mean ± SEM; **P=0.003, ****P<0.0001, ns, not significant, Two-way ANOVA.

In conjunction with previous studies (10), the results above support a potential role of activated and IFN-γ producing T-cells in eliciting the response to combined vanadate/VSVΔ51 therapy. We next sought to investigate the impact of VS treatment in combination with VSVΔ51 on viral- and tumor antigen-specific immune responses. We assessed the antigen specific immune response against peptides for gp70 (the immunodominant antigen in CT26WT tumor model and a common murine tumor-associated antigen that is highly expressed) and VSV N following combined therapy or controls using an IFN-γ ELISPOT assay. Fourteen days post treatment, splenocytes were isolated from mice and then stimulated *ex vivo* with gp70 or VSV N peptides (**Figure 2A**). A significant increase in the number of IFN-γ producing splenocytes reacting to the peptide gp70 was detected in the group that received VS+VSVΔ51 when compared with monotherapy alone (**Figure 2B**). As expected, treatment with VSVΔ51 alone induced an antiviral immune response measured by the reaction against the VSV N peptide in comparison with the combination treatment group (**Figure 2B**). However, we did detect a response against the DMSO control in the VSVΔ51 group, suggesting there could be non-specific activation owing to the virus treatment. Altogether, these results suggest that VS/VSVΔ51 combination therapy can improve the tumor antigen-specific T-cell response.

### 2.3 Enhanced antigenicity driven by vanadium implicates a cancer cell autonomous mechanism

In previous studies, we showed that the treatment of cancer cells with vanadate/VSVΔ51 leads to profound changes in gene expression profiles in infected cancer cells and in particular, the up-regulation of multiple cytokines and chemokines associated to response to type II IFN (10). This profile is corroborated from cytokine profiles in **Figure 1**. However, given that cytokine profiles measured in the blood are representative of systemic responses, it is not possible to discern the specific impact of VS+VSVΔ51 treatment on cancer cells from its potential impact on immune cells.

**Figure 3:**
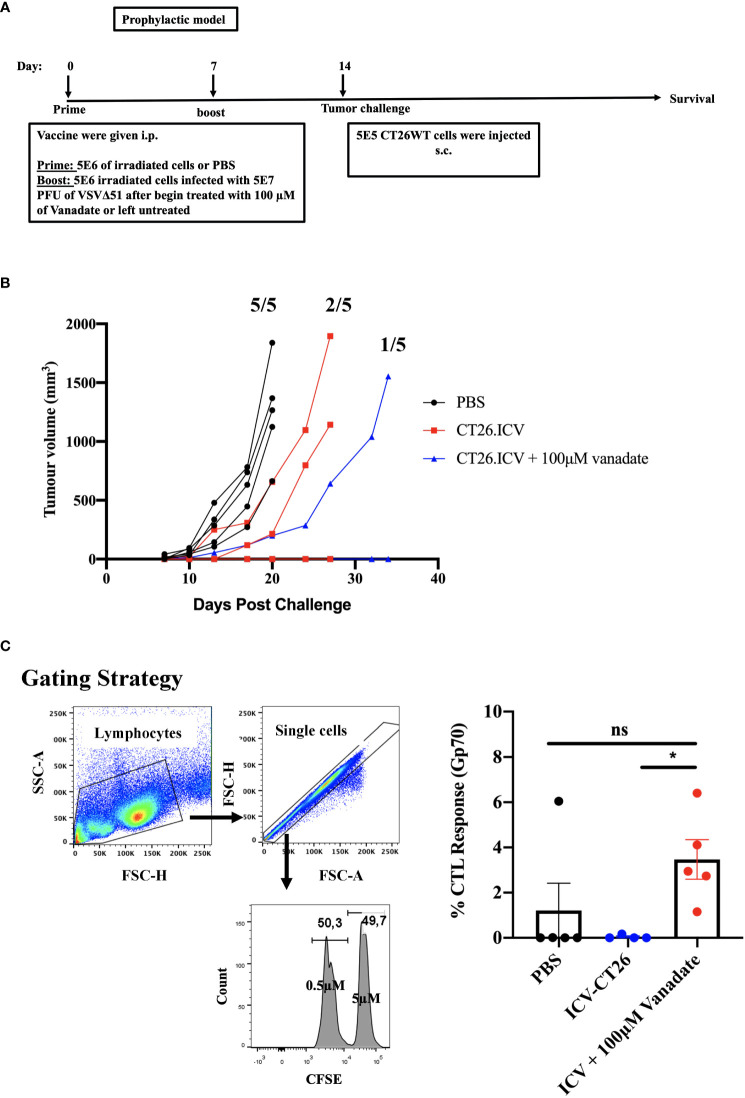
VS enhances irradiated infected cell vaccine antigenicity. **(A)** Schematic representation of the treatment schedule of prophylactic vaccine, CT26WT-tumor bearing mice injected with 5E6 irradiated CT26WT cells i.p. at day 0, and 7 days following that 5E6 irradiated CT26WT cells infected with 5E7 PFU VSVΔ51 with and without vanadate were injected i.p. At day 14, CT26WT cells were implanted s.c. **(B)** Tumor volume of each individual mouse measured over time (n=5 per condition). Fractions indicate the proportion of mice that developed tumors in each group. **(C)** following the same therapeutic regiment described in A, 7 days post the last treatment, splenocytes from immunized mice were harvested eighteen hours after injecting an equal number of CFSE_Hi_ cells (that were labelled with 5µM CSFE) loaded with gp70 peptide and CFSE_Lo_ cells (labelled with 0.5µM CSFE) unloaded with peptide, in equal proportions of 2E7/200μl total splenocytes intravenously (i.v.). The proportion of CFSE_Hi_ and CFSE_Lo_ was quantified by flow cytometry by first gating for lymphocytes using forward scatter (FSC) and side scatter (SSC) followed by single-cell gating, the CSFE_Hi_ and CFSE_Lo_ populations. The graph shows the percentage of specific killing against Gp70-CFSE-stained targeted cells compared to control groups. Mean ± SEM; * P = 0.02, One-way ANOVA, n = 5 per conditions. ns, not significant.

To better understand whether cancer-cell autonomous mechanisms could contribute to the improved tumor antigen responses observed in the vanadate/VSVΔ51 conditions, we employed a prophylactic infected cancer cell vaccination (ICV) model to isolate the impact of vanadate/VSVΔ51 treatment *in vitro* on the immunogenicity of the same *in vivo*. As previously published (20, 21), ICVs involve irradiation of cancer cells, followed by infection with an OV *in vitro*. Administration of ICV elicits both antitumor and anti-viral immune responses. The antitumor response can be further boosted when an initial prime with irradiated cancer cells alone is administered 7 days prior to the ICV (**Figure 3**) and can be measured among others by looking at the impact on tumor growth following challenge with the same cancer cells used to create the ICV.

**Figure 4:**
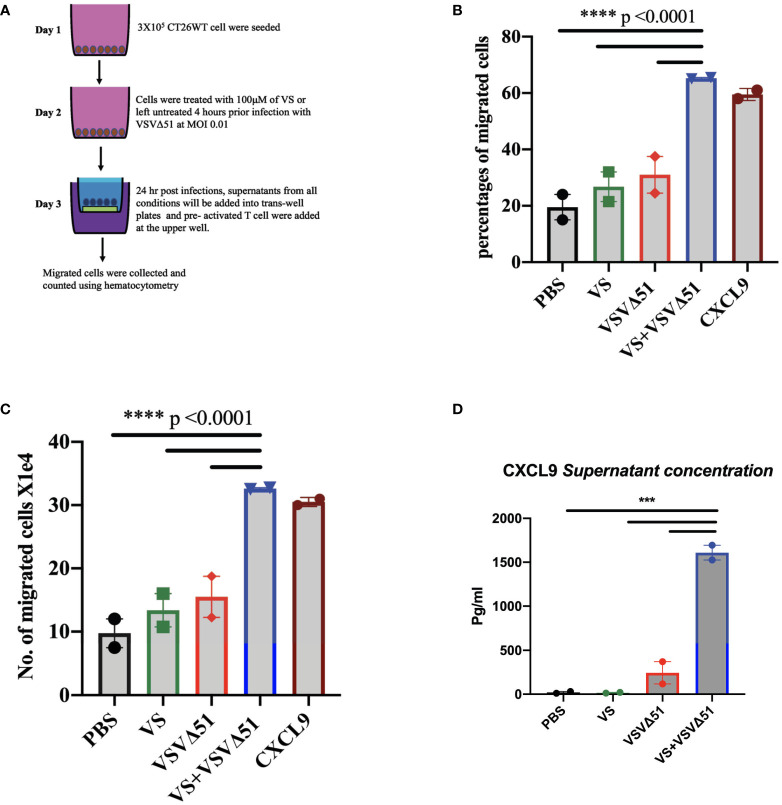
Vanadyl sulfate in combination with VSVΔ51 increases the production of T-cell chemoattractant and T-cell migration *in vitro*. **(A)** Tissue culture supernatants were collected after treating CT26WT cells with vanadyl sulfate at a concentration of 100μM for 4 hours prior infection with VSVΔ51 at MOI 0.01 or with PBS, vanadyl sulfate alone or VSVΔ51 alone for 24 hour and subsequently used for the assessment of chemokines or for performing chemotaxis assays (compared to CXCL9 control). Chemotaxis of T cells was assessed using a Trans-well system. **(B)** Migration percentage was calculated as (total number T cells in bottom chamber/total number T cell input) x 100. **(C)** The Number of migrated cells X10^4^ with the mean of the duplicate for each condition. Mean ± SEM; ****P<0.0001, Two-way ANOVA. Representative data of the average of 2 independent experiments, n = 2 per condition. **(D)** The supernatant concentration of CXCL-9, graph shows the pg/ml. Mean ± SEM; n = 2 per condition, *** P = 0.001, Two-way ANOVA.

We generated ICV preparations using 5E6 CT26WT cells infected with 5E7 PFU of VSVΔ51 (MOI=10), with or without 100μM vanadate. After collection, these were administered to Balb/C mice previously primed with irradiated CT26WT cells (5E6) intraperitoneally (i.p). On day 7 post ICV administration, mice were challenged with 5E5 CT26WT cells subcutaneously (s.c.). In **Figure 3A**, we monitored tumor progression following the ICV treatments. We observed that mice prophylactically treated with irradiated cells infected with VSVΔ51 in combination with vanadate developed fewer tumors with more delayed kinetics compared to the group receiving ICV made using VSVΔ51 alone (**Figure 3B**). In addition, ICV made using vanadate further stimulated specific killing against targeted cells loaded with gp70 peptides, as measured by *in vivo* CTL assay in vaccinated mice (p=0.0219 comparing ICV group with ICV+Vanadate group, **Figure 3C**). This altogether suggests that vanadate treatment of cancer cells in conjunction with VSVΔ51 infection promotes their inherent immunogenicity, independently of a direct impact of vanadate on immune cells. Given we have previously reported the increased expression of type II IFN response-induced chemo-attractants such as CXCL9 and CXCL10 following vanadate/VSVΔ51 co-treatment, we looked at whether VS+VSVΔ51 co-treatment could promote the recruitment of T-cells in a chemotaxis assay. As illustrated in **Figure 4**, cultured CT26WT cells were treated with PBS, VS 100μM, VSVΔ51 (multiplicity of infection (MOI)=0.01) or the combination of VS+VSVΔ51. Supernatants were collected 24h later and added to the lower chamber of Trans-well plates with 5-μm pores. A condition with CXCL9 cytokine (1 μg/ml) was included as a positive control. Mouse T-cells isolated from splenocytes were activated using anti-CD3/CD28 beads and IL-2 and then added to the top chamber. Activated cells were allowed to migrate to the bottom well containing supernatants for a period of 24h. The results of this chemotaxis assay are shown in **Figure 4** and demonstrate that supernatants from VSVΔ51-infected cells co-treated with VS significantly increase the migration of activated T-cells compared to single treatments, to levels exceeding the CXCL9 positive control (**Figures 4B, C**). The CXCL-9 concentration in the supernatants used in the migration assay was measured by ELISA. As shown in **Figure 4D**, treatment with VS+VSVΔ51 substantially increased the secretion of CXCL-9 compared to single treatments. Altogether, these data support the hypothesis that treatment of cancer cells with vanadium during infection increases their immunogenicity profile and promotes the migration of activated T-cells to the tumor.

### 2.4 VS in combination of VSVΔ51 encoding IL-12 further improves therapeutic efficacy

The results above implicate a role of T-cells in eliciting the enhanced therapeutic effects of VS+VSVΔ51 treatment and that is also associated with up-regulation in the secretion of pro-inflammatory cytokines and chemokines; however, the overall survival is still 20% in the CT26WT tumor model (**Figure S3**). We therefore considered whether therapeutic efficacy could be further enhanced by genetically encoding complementary cytokines in VSVΔ51.

**Figure 5:**
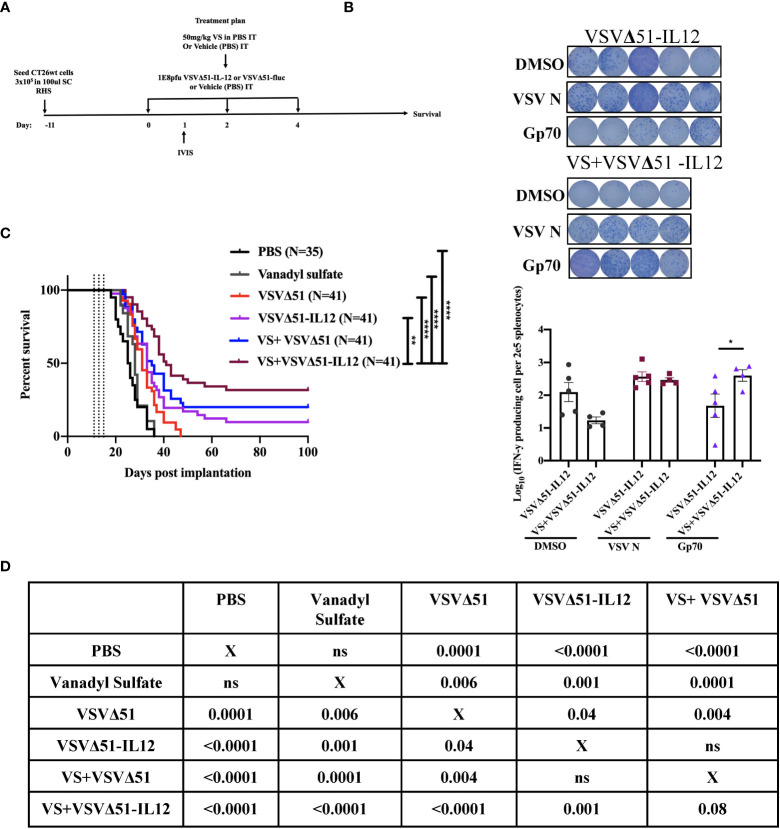
Vanadyl sulfate in combination with VSVΔ51 encoding/expressing IL-12 improves the therapeutic efficacy in CT26WT-tumor model **(A)** Schematic representation of the treatment schedule: CT26WT-tumor-bearing mice received a total of three dose delivered intratumorally of vanadyl sulfate (50mg/kg) and VSVΔ51 expressing IL-12 (1E8 PFU) or with PBS, vanadyl sulfate (50mg/kg), or VSVΔ51 expressing IL-12 (1E8 PFU) alone over 5 days. **(B)** Survival was monitored over time, the percentage of survival was included for each condition, Long rank (Mantel-Cox) test indicated the significance as shown in table below. Survival data represents an aggregate of 3 independent studies. **(C)** as part of **Figure 2**, two additional groups received three intratumorally vanadyl sulfate (50mg/kg) and VSVΔ51 expressing IL-12 or monotreatment injections over 5 days. representative data from ELISPOT assay and Graph shows quantification of log_10_ of the number of IFN-γ producing cells per 2E5 splenocytes. Mean ± SEM; * P=0.01, ** P=0.003, ****P<0.0001, Two-way ANOVA. **(D)** Table shows the significance values using Long rank (Mantel-Cox), * P=0.01, ** P=0.003, ****P<0.0001.

**Figure 6:**
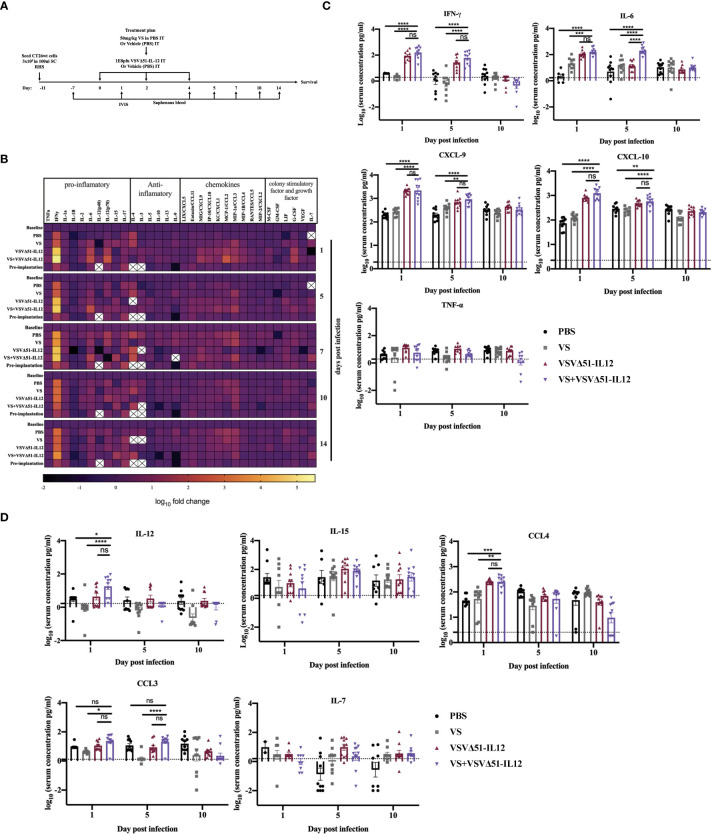
VS+VSVΔ51 encoding/ expressing IL12 co-treatment further induces the upregulation of the secretion of pro-inflammatory chemokines and cytokines 1 day and 5 days post the treatment. **(A)** CT26WTtumor bearing mice received a total of three doses delivered intratumorally of vanadyl sulfate (50mg/kg) and VSVΔ51 expressing IL-12 (1E8 PFU) or with PBS, vanadyl sulfate (50mg/ kg), or VSVΔ51 expressing IL-12 (1E8 PFU) alone over 5 days. **(B)** Heatmap of the log_10_ fold change per condition of pooled sera, normalized to day 7 post implantation as baseline for all the tested time points. **(C, D)** The concentration of cytokines and chemokines pg/ml for each group for each individual mouse (n=10 per condition) was presented in each graph. Data transformed into Log_10_, line indicated the detection concentration, Mean ± SEM; * P = 0.01, ** P = 0.003, *** P = 0.001, ****P < 0.0001, Two-way ANOVA. ns=not significant

As a first candidate and proof of concept, we evaluated the impact of encoding IL-12 within VSVΔ51. IL-12 is produced by dendritic cells (DC) in response to IFN-γ secretion to enhance the activity of CD8+ cytotoxic T-cells (CTL) as well as Natural Killer cells (NK) (22, 23). Further, IL-12 is thought to be a positive regulator of IFN-γ secretion. Many OVs such as Maraba-MG-1, Adenovirus, and HSV-1 have been engineered to express IL-12, leading to improved antitumor effects in numerous murine carcinoma models by promoting both the activation of T cells and NK cells (24, 25). As a first step, multi-step and single-step viral growth kinetics were determined in CT26WT cells, and the inclusion of IL-12 as transgene had no impact on virus replication kinetics compared with parental virus VSVΔ51encoding firefly luciferase (**Figure S4A**). In addition, IL12 secretion was confirmed 24 hr following viral infection compared with the parental virus (around 6000pg/ml) (**Figure S4B**). To validate the impact of IL-12 in our system, we performed a similar therapeutic regimen as shown in **Figure 1A** with VSVΔ51 encoding/expressing IL12 (**Figure 5A**) and then monitored tumor progression and survival following the treatment. Combination treatment was well-tolerated although we did observe a slight decrease in the body weight of mice treated with VSVΔ51 encoding/expressing IL12 during the week of the treatment followed by quick recovery within the following week (**Figure S1**). Our data demonstrate that approximately 40% of the group receiving VS in combination with VSVΔ51-IL-12 survived compared to 20% in the VS+VSVΔ51 condition, and only 10% in the group receiving the VSVΔ51 expressing IL-12 alone (**Figures 5B, D**). These mice also showed rejected tumors after re-challenge with CT26WT cells. The inclusion of IL-12 to VSVΔ51 in combination with VS also further enhanced the antigen specific immune response against the gp70 peptide (**Figure 5C**); however, there was no apparent improvement in comparison with VS+VSVΔ51 treatment (compare **Figures 2B and 5C**). Cytokine profiling by multiplex assay (**Figure 6A**) revealed a trend towards increasing systemic IL-12 with VSVΔ51-IL12 and further with VS+VSVΔ51-IL12, which was the only condition showing a statistically significant increase in IL-12 relative to PBS (p-value=0.023, **Figure S5**) (**Figures 6B, C, D**). In contrast with results obtained in **Figure 1**, VS+VSVΔ51-IL12 co-treatment did not elicit a further increase in the levels of IFN-γ compared to VSVΔ51-IL12 (**Figures 6B, C**); however, compared to **Figure 1D**, it is apparent that when viruses were used alone, IFN-γ reached higher levels on day 1 with VSVΔ51-IL12 compared to VSVΔ51 (more than 200 pg/ml vs. around 100 pg/ml). On the other hand and consistent with **Figure 1C**, levels of IL-6 were significantly higher at day 5 following VS+VSVΔ51-IL12 combination treatment compared to the monotherapies (**Figures 6B, C**). Taken together, these data provide compelling evidence that further improvements in therapeutic efficacy can be achieved through genetic complementation by encoding cytokines such as IL-12 that are not otherwise up-regulated by VS/VSVΔ51 co-treatment.

## 3 Discussion

In this study, we found evidence that VS+VSVΔ51 combination treatment leads to an increase in the *in vivo* secretion of type II-IFN related cytokines, including IFN-γ and IL-6. Complementing previous studies showing increased infiltration of activated T-cells in tumors from combination-treatment responsive animals (10), we observed an increase in the number of tumor antigen-specific T-cells in the spleen. These findings were supported by the notion that treatment of cancer cells with vanadate during infection with VSVΔ51 can increase the immunogenicity of cancer cells, which is explained in part by a cancer cell autonomous driven mechanism as confirmed by the ICV and *in vitro* chemotaxis experiments.

The role of IFN-γ in the context of the antitumor immune response remains unclear based on the literature. For example, it has been reported that antigen-specific T-cells increase IFN-γ concentration in tumor tissue, which facilitates tumor clearance (26). However, IFN-γ also induces the expression of PD-L1 on lymphatic endothelial cells, negatively impacting cytotoxic T-cell activity and migration from the peritumoral space to the tumor microenvironment (27). High levels of IFN-γ can also induce apoptosis in tumor-specific T-cells and compromise antitumor immunity (28, 29). On the other hand, recent reports have suggested that tumor IFN-γ signatures are associated with greater response to immune checkpoint blockade (30), other studies have also shown that encoding IFN-γ from OVs like VSVΔ51 can improve therapeutic benefit (31).

While we observed elevated IFN-γ at early timepoints following combination treatments with VS and VSVΔ51, we did not see further increases in IFN-γ secretion when including IL-12 as a transgene, despite nearly doubling the rate of complete remissions (from 20% to 40%). While we cannot exclude the possibility that IFN-γ may negatively impact the effect of VS and VSVΔ51, this suggests that other factors must contribute to curative responses. One hypothesis that warrants further study is that an increased chemotaxis of activated and tumor antigen-specific cytotoxic T-cells to the tumor, potentially *via* chemokines such as CXCL9 and CXCL10. Indeed, this is supported by our previous studies showing more substantially elevated levels of activated T-cell in tumors in the process of regression in comparison to non-responders (10).

Interestingly, we found that IL-6 is consistently and significantly increased with VS combination treatments using either VSVΔ51 or VSVΔ51-IL12 during the first 5 days post treatment. IL-6 is an NF-kB-regulated cytokine known to inhibit T-cell apoptosis and prevent regulatory T-cell differentiation, but also plays an essential role in the differentiation of monocytes to macrophages (32). As previously reported by Fisher et al. and others, IL-6 can further increase naïve and central memory T cell trafficking to the tumor (32–34). The elevated levels of IL-6 observed peaking at 5 days post treatment in this study are therefore consistent with an increase in tumor-antigen-specific T-cell response (**Figure 2**). This observation is also consistent with previous *in vitro* studies, implicating EGFR pathway activation as a molecular mechanism of vanadium compounds, leading to simultaneous downregulation of STAT2 and hyperactivation of both STAT1 and NF-kB (19). On the other hand, IL-6 is also suspected to play a role in tumour development (35) and some groups have found a correlation between high circulating levels of IL-6 and poor prognosis in cancer patients (36). However, in this study we observed only transiently increased levels of IL-6 returning to normal levels after day 5. The observation of complete tumor regression in this context suggests that at least transient up-regulation of IL-6 may be beneficial to generate robust anti-tumour responses.

We observed increased gp70 specific T-cells in the spleen of animals treated with combination regimen. While there was also a detectable increase in anti-viral immune response in the combination treatment, its magnitude was subdued compared to the increase in anti-gp70 response (**Figure 2B**). One possibility is that the tumor-specific antigen response is in effect a memory response since the tumors were implanted over a week prior to the first virus treatment. Supporting this idea, we find that in the ICV model, antitumor responses are significantly improved following an initial prime with irradiated CT26WT cells, followed by a boost with infected-irradiated cells a week or more later (**Figure 3C**). If validated, this hypothesis may further suggest a preferential impact of vanadate on memory T-cells as opposed to naïve T-cells. Further studies will be required to address this question.

In this study, IL-12 was selected for provision in cis with VSVΔ51 to enhance the therapeutic effect in combination with VS in part because it was previously shown to provide therapeutic benefit with other OVs (37–40). While it is challenging to rationalize the choice of transgene to enhance OV activity based on cytokine profiling owing to the complexity and kinetics of cytokine signaling networks, cytokine profiling in this case was helpful to rationalize IL-12 as a choice to further improve activity. IL-12 is known to drive IFN-γ expression which led us to hypothesize that inclusion of the IL-12 transgene may further drive upwards IFN-γ secretion when included within the virus. However, underscoring the complexity of cytokine networks, we did not observe increased IFN-γ secretion upon inclusion of IL-12 within VSVΔ51, despite observing improved therapeutic effects. While significantly increased at day 1 following treatment in the VS+VSVΔ51-IL12 condition compared to PBS, we found that IL-12 levels in the blood remained low (**Figure 6**). However, high levels of replication in the tumor as measured with VSVΔ51-Fluc in combination with VS (**Figure 1**), may suggest that a localized IL-12 delivery safely generates a systemic effect that induces an antitumor immunity that benefits a locally initiated immune response (41–43). It would be interesting to determine whether supplementation of cytokines and chemokines not detected to be significantly up-regulated but that could play a complementary role could further enhance activity in combination with VS when encoded in the virus either alone or potentially in combination with IL-12.

In conclusion, our results add to a growing body of evidence demonstrating the potential of vanadium compounds as viral enhancers in combination with OVs to improve their therapeutic efficacy. This study provides a new framework for understanding the immunological impacts of vanadium-VSV combinations, and provide a path forward for therapeutic regimen optimization, exemplified by the combination of VS and VSVΔ51-IL12.

## 4 Material and methods

### 4.1 Drugs

The vanadium-based compounds used in this study were sodium orthovanadate and vanadyl sulfate. Sodium orthovanadate was used for all ICV experiments (Na_3_VO_4_, Sigma-Aldrich, Cat# 450243) and was dissolved in water and pH adjusted to 10 at 100μM. Where otherwise indicated, vanadyl sulfate at 50 mg/kg (VOSO_4_, Sigma-Aldrich, Cat# 204862) dissolved in PBS was used for *in vivo* experiments whereas 100μM was used for *in vitro* experiments.

### 4.2 Cell lines

CT26WT (murine colon carcinoma, Cat.CRL-2638) was obtained from the American Type Culture Collection (ATCC). These cells were cultured in HyQ high-glucose Dulbecco’s modified Eagle’s medium (DMEM; HyClone Cat#10-013) supplemented with 1% (v/v) penicillin-streptomycin (Gibco Life Technologies, Waltham, MA), and 10% (v/v) serum composed of 3-parts HyClone newborn calf serum (Thermo Fisher, Cat# SH3011803) and 1-part Fetal Bovine Serum (Gibco, Cat# 12483020). All cells were incubated at 37C in 5% CO2 humidified incubator and regularly tested to ensure they are free of mycoplasma contamination.

### 4.3 Viruses

#### 4.3.1 Rhabdovirus

VSVΔ51-Fluc: The Indiana serotype of VSV used throughout this study was propagated in Vero cells and purified on 5-50% Optiprep (Sigma-Aldrich, St. Louis, MO). VSVΔ51 expressing firefly luciferase are recombinant derivatives of VSVΔ51 described previously (10).

VSVΔ51 encoding/expressing IL-12: VSVΔ51 encoding/expressing IL-12 was generated in the laboratory of Dr. Rebecca Auer using PCR amplified from pORF-mIL12 [IL12elasti(p35:p40); *In vivo*Gen] *(* 20). Virus was produced and purified on 5-50% Optiprep (Sigma-Aldrich, St. Louis, MO) in our laboratory.

### 4.4 Mouse tumor model

Six-week-old female BALB/c mice obtained from Charles River Laboratories (Senneville, Canada) were implanted with subcutaneous tumors by injecting 3E5 syngeneic CT26WT cells, suspended in 100μl serum-free DMEM. Eleven days post implantation when tumor reached a volume of 100 mm^3^, mice were treated intratumorally vanadyl sulfate (dissolved in PBS) at 50mg/kg in 50μl or 50μl of PBS. Four hours later, mice were treated intratumorally with 1E8 plaque forming units (PFU) in 25μl PBS of the indicated viruses as described previously (10). Tumor sizes were measured three times a week using electronic calipers. Tumor volumes were calculated based on this equation (length^2^ X width)/2. All *in vivo* experiments were performed in accordance with the University of Ottawa Animal Care and Veterinary service guidelines for animal care under the protocol OHRIe-2265-R5 and OHRI-2265-R4 A2.

### 4.5 Blood and serum sample processing

For blood samples, saphenous bleeds were performed in one of the legs and blood was collected in tubes containing Serum-Gel (Sarstedt Inc, Fisher Scientific, Cat. NC9315741) to allow serum separation by centrifugation (14 000 rpm) for 10 mins using Eppendorf centrifuge 5418R, then serum was used for measuring cytokine and chemokines. Serum samples were stored at –80C after collection.

### 4.6 Multiplex assay

Serum from blood samples was collected at day 7 days post implantation as a baseline, and 24 hours to 14 days post treatment. Serum was then added in duplicate for each sample in a 96-well plate, and magnetic microspheres premixed with 32-plex beads were added according to the manufacturer’s recommendation (MILLIPLEX MAP Mouse Cytokine/Chemokine Magnetic Bead Panel - Premixed 32 Plex - Immunology Multiplex Assay, Millipore Sigma, Cat# MCYTMAG-70K-PX32) and incubated overnight at 4°C. A biotinylated detection antibody was added, followed by streptavidin-PE conjugate antibody incubated for 1 hr at room temperature. Plates were run in the Luminex^®^ analyzer (MAGPIX^®^), using a CCD-based analysis that captures and detects components with the speed and efficiency of magnetic beads. All measurements were performed using a MAGPIX instrument (Luminex, Ottawa, CA), following the manufacturer’s instructions. Data were collected using xPonent software (Luminex, Version 4.2), log transformed, and analyzed as described below in the statistical analysis section.

### 4.7 Splenocyte processing

Spleens were processed into single cell suspension by first transferring the spleen into sterile 70μm cell strainers on 50 ml conical tubes. Then, spleens were softly minced by pressing against the flat side of a syringe plunger in a circular motion. Cell strainers were washed with 5 ml of R 10 media. Tubes containing the cell suspension were topped up with media and spun at 300 g for 5 mins. Carefully, supernatants were removed without disturbing the pellet and cells resuspended by gently tapping before adding the ACK (Ammonium-Chloride-Potassium) lysing buffer (Gibco™, Cat# A1049201) for lysing red blood cells in samples for 5 mins. Cells were then washed and resuspended with R 10 media.

### 4.8 Peptides for *ex vivo* stimulation

Peptides used for *ex vivo* stimulation were gp70 tumor peptide: H-2Ld SPSYVYHQF (MBL International Corporation, Cat# SPM521) and VSV N virus peptide: H-2Ld MPYLIDFGL (CanPeptide Inc, custom peptide)

### 4.9 Mouse IFN-γ single-color enzyme-linked immune absorbent spot

Cells from spleens were processed as previously described and then resuspended in CTL media (provided with the ELISPOT kit, Immunospot, Cat# mIFNgp-1M/5) in 2E5 cells/100μl before being added to IFN-γ pre-coated plates (Immunospot, Cat# mIFNgp-1M/5) containing 100μl/well of each stimulant including either gp70 or VSV N peptides at a final concentration of 10 μM as well as unstimulated control (equivalent amount of DMSO). PMA ((Phorbol 12-myristate 13-acetate, Sigma-Aldrich, Cat# P8139)/ionomycin (Sigma-Aldric Cat# I9657) for pooled samples were used as a positive control at 2μg/ml. Plates were then incubated at 37°C for 20 hours. Following that, ELISPOT assays were developed following the manufacturer’s protocol. Plates were scanned and spots were counted using CTL S6 ImmunoSpot analyzer.

### 4.10 Infected cell vaccine preparation

CT26WT cells were harvested and washed twice with Phosphate Buffered Saline (PBS; Corning, Manassas, VA), and then resuspended in DMEM at 5E7 cells/mL (± 10%). Cells were then irradiated in a Pantak HF320 X-Ray machine (OHRI, Ottawa, CA) for 60 Gy before infection with VSVΔ51 at a Multiplicity of Infection (MOI) of 10, for 2 hours in rotation before *i.p.* delivery (5 x 10^6^ cells; 5E7 plaque forming units (PFU) of virus/mouse). Irradiated CT26WT cells (*irr)* were prepared similarly without the infection step. Cells were treated with 100μM vanadate before infecting them for some conditions.

### 4.11 Infected cell vaccine mouse model

In prophylactic treatment model: BALB/c mice were treated with 5E6 irradiated CT26WT cells on day 0 intraperitoneally (i.p.), followed by treatment with ICV on day 7 i.p (5E6 irradiated CT26WT cells infected with 5E7 PFU of VSVΔ51). At day 14 mice were challenged with 5E5 CT26WT cells s.c.

### 4.12 *In vivo* CTL assay

Donor splenocytes from naïve Balb/c mice were labelled with either a high (5μM) or low (0.5μM) concentration of carboxyfluorescein succinimidyl ester (CFSE; ThermoFisher Scientific, Waltham, MA, Cat# 65-0850-84) for 10 minutes at 37C. Each tube was then topped up with R 10 media and spun down for 5 minutes at 1500 rpm to remove excess dye. The CFSE-high (CFSE_Hi_) population of naïve splenocytes is subsequently peptide-loaded with exogenous tumour gp70 tumour peptide (H2Ld-restricted, SPSYVYHQF; MBL International, Woburn, MA) at a concentration of 5μM for 45 minutes at 37°C rotated every 15 mins, whereas the CFSE-low (CFSE_Lo_) population was left unloaded. Cells were washed with R 10 media to remove excess peptides and spun down for 5 minutes at 1500 rpm, then re-suspended in serum-free media. An equal number of CFSE_Hi_ and CFSE_Lo_ populations were combined in equal proportions of 1E7 cells/100μl each in serum-free media and a total of 2E7/200μl cells were administered intravenously (i.v.) to previously vaccinated mice. Eighteen hours later, vaccinated mice were sacrificed and splenocytes were harvested. Flow cytometry was used in order to assess the relative elimination of the tumour antigen-loaded CFSE_Hi_ population compared to control groups. Percent specific lysis was calculated based on this equation Percent specific lysis= [1-[(CFSE_Lo_/CFSE_Hi_ in PBS mice)/(CFSE_Lo_/CFSE_Hi_ in treated mice)]]x100.

### 4.13 Migration/chemotaxis assay

CT26WT cells were pretreated with 100 μM VS or PBS for 4 hours followed by viral infection with VSVΔ51 at MOI 0.01 or mock infection. 24 hours later, cultured supernatants were collected and subsequently used for the assessment of chemokines or for performing chemotaxis assays. Chemotaxis of T cells was assessed using a Trans-well system as described previously (20). Briefly, 700μl of conditioned media from CT26WT cell cultures described above was added to the lower chamber of Trans-well plates with 5-µm pores (Costar, Corning, sigma-Aldrich, Cat# CLS3421-48EA). 5E5 activated CD3+ T cells from naïve spleen were purified by negative selection using EasySep™ mouse isolation T cell kit (Stemcell technologies, Cat# 19851) and then activated with 2μg of anti-CD3/CD28 in a precoated plate (BD Pharmingen™, CD3 Cat# 550275 and CD28 Cat# 553295) supplemented with 2ng/ml of murine recombinant IL-2 (PeproTech, Cat# 212-12) for 2 days, were added to the upper chamber, and plates were incubated for 24 hours at 37°C. After 24 hours, cells in the lower chambers were collected, stained with trypan blue and counted with a hemacytometer. Migration percentage was calculated as follows: total number of T cells in bottom chamber/total number of T cell input) x 100 (20).

### 4.14 Viral growth curves

3E5 of CT26WT Cells were seeded in 24 well plates for overnight confluency. Cells were then inoculated with VSVΔ51-Fluc at an MOI of 0.01 (multi-step growth curve) or 1.0 (single-step growth curve). Cells infected at MOI 1.0 were incubated for 60 minutes, followed by washing and replenishing with fresh medium. Cells were incubated up to 48 hours post infection (hpi), with 200 µl of supernatant collected and frozen at -80°C at the following timepoints: 0, 4, 8, 12, 24, 32, 48 hpi. Viral titer in collected supernatant was quantified by high-throughput tittering using a standard plaque assay.

### 4.15 Plaque assay

For virus titration using standard plaque assay, Vero cells African green monkey kidney cells, Cat# CCL-81) obtained from the American Type Culture Collection (ATCC) were seeded into 12-well plates at a final density of 3E5 cells per well. Infectious supernatants were serially diluted using serum-free DMEM, transferred (500µL/well) onto Vero cells and incubated at 37°C, 5% CO2 for 45 minutes, following which media was removed and replaced with 1ml/well of an agarose overlay (1:1 ratio of 1% agarose mixed with 2X DMEM containing 20% FBS). After a 24 hours incubation, plaques were fixed with methanol: glacial acetic acid in a 3:1 ratio for a minimum of 1 hour, then stained for 30 minutes with a Coomassie Blue solution (4 g Coomassie Brilliant Blue R (Sigma, Cat# B0149), 800 ml methanol, 400 ml acetic acid and 2800 ml distilled water) to visualize and count plaques.

### 4.16 IL-12 p70 Enzyme-linked immunosorbent assay

Supernatants from infected CT26WT cells with VSVΔ51 encoding Fluc or IL12 at MOI 0.1 were collected 24 hours post infection. The secretion of IL12 was measured using mouse IL-12 p70 Quantikine ELISA (Mouse IL-12 p70 Quantikine ELISA Kit, R&D system, Cat#M1270) following the manufacturer’s instructions. Absorbance values at 450nM were measured on a Multiskan Ascent Microplate Reader (MXT Lab System) and corrected for plate imperfections at 540. Data were analyzed using Prism.

### 4.17 CXCL9 Enzyme-linked immunosorbent assay

CT26WT cells were pretreated with 100 μM VS or PBS for 4 hours followed by viral infection with VSVΔ51 at MOI 0.01 or mock infection. 24 hours later, cultured supernatants were collected and the concentration of CXCL9 was measured using mouse MIG/CXCL9 ELISA Kit (ELM-MIG, RayBiotech, Cat# ELM-MIG-2) following the manufacturer’s instructions. Absorbance values at 450nM were measured on a Multiskan Ascent Microplate Reader (MXT Lab System) and corrected for plate imperfections at 540. Data were analyzed using Prism.

### 4.18 Statistical analysis

Statistical significance was calculated using Student’s t test or one-way or two-way ANOVA tests with Sidak or Dunnett’s multiple correction test was applied when groups were split on multiple independent variables and also using Tukey’s multiple comparison test. Mantel-Cox log rank test was used to determine significant differences in plots for survival studies. Error bars represent standard error of the mean. Significance is based on a p value <0.05. Statistical analyses were performed using Graph-Pad Prism 8.0 (GraphPad software, LLC) and Excel. For multiplex data, the analysis software is milliplex analyst, version 5.1.0.0. VigeneTech Inc using 5 parameter curve fitting (log scale). Data were then transformed to log_10_ then normalized to the baseline values for each cytokines/chemokines for each time point to quantify the fold changes. For individual response, data transformed to log_10_ as plotted in the graphs to be able to see the significant difference. For ELISPOT analysis software is ImmunoSpot version 7.0.20.0, Flow cytometry was performed at the University of Ottawa’s Flow Cytometry Core Facility using the BD LSR Fortessa (BD) or BD Celesta (BD) Flow Cytometers. Analysis of flow data was conducted using FlowJo v10 (FlowJo, LLC, Ashland, OR). For all analyses, *p < 0.05, 872 **p < 0.01, ***p < 0.001, ****p < 0.0001; n.s. = not significant.

## Data availability statement

The original contributions presented in the study are included in the article/**Supplementary Material**. Further inquiries can be directed to the corresponding author.

## Ethics statement

All *in vivo* experiments were performed in accordance with the University of Ottawa Animal Care and Veterinary service guidelines for animal care under the protocol OHRIe-2265-R5 and OHRI-2265-R4 A2.

## Author contributions

Conceptualization, NA and J-SD; Methodology, NA conducted the *in vitro* and *ex vivo* experiments. NA and AC performed all *in vivo* experiments. RoA contributed to the IL-12 related *in vivo* survival experiments. AJ, ZT, and GM assisted with tissue processing. OV and SK contributed to the experimental design and performed the ICV experiments. NA and J-SD designed the experiments, performed data analyses, and wrote the paper. RoA, NF, and DS, helped with experimental design. NA, DS, RoA, AJ, ZT, and J-SD reviewed the paper; Funding Acquisition, J-SD; Supervision, J-SD. All authors contributed to the article and approved the submitted version.

## Funding

J-SD is supported by funding from the Terry Fox Research Institute, TFF-122868 (J-SD) Canadian Institutes of Health Research, grant INI-147824, Canadian Institutes of Health Research, grant #705952, a Canadian Cancer Society grant supported by the Lotte & John Hecht Memorial Foundation, grant #703014 (J-SD). NA is supported by a scholarship from King Faisal Specialist Hospital and Research Center, Riyadh, Saudi Arabia (KFSHRC) representative by Saudi Arabian Cultural Bureau in Canada (SACB). ZT is funded by NSERC Alexander Graham Bell Canada Graduate Scholarship, Ontario Graduate Scholarship, and Mitacs CanPRIME Accelerate fellowship. AJ is supported by the MITACS Accelerate Canadian Partnership in Immunotherapy Manufacturing Excellence PhD Internships. OV was supported by a CIHR Master’s Award.

## Acknowledgments

The Authors thank Almohand Alkayyal for his generous help with optimizing the migration assay, Alexandra Acal for her assistance with some experiments, and Stephanie Burke Schinkel for her generous help with the MAGPIX instrument. The Authors also thank Dr. Debbie C. Crans for her helpful discussion surrounding vanadium chemistry

## Conflict of interest

J-SD is co-inventor on a patent covering the use of vanadium compounds as enhancers of RNA viruses and holds shares in Virica Biotech and Virano Therapeutics that hold licenses to this intellectual property.

The remaining authors declare that the research was conducted in the absence of any commercial or financial relationships that could be construed as a potential conflict of interest.

## Publisher’s note

All claims expressed in this article are solely those of the authors and do not necessarily represent those of their affiliated organizations, or those of the publisher, the editors and the reviewers. Any product that may be evaluated in this article, or claim that may be made by its manufacturer, is not guaranteed or endorsed by the publisher.
