# Supplementary material for: Vanadyl sulfate-enhanced oncolytic virus immunotherapy mediates the antitumor immune response by upregulating the secretion of pro-inflammatory cytokines and chemokines
Source: Front Immunol. 2022 Nov 28;13:1032356. doi: 10.3389/fimmu.2022.1032356 (PMC9749062; doi:10.3389/fimmu.2022.1032356)
Supplement: Supplementary file 1 [file DataSheet_1.pdf]

Supplementary materials

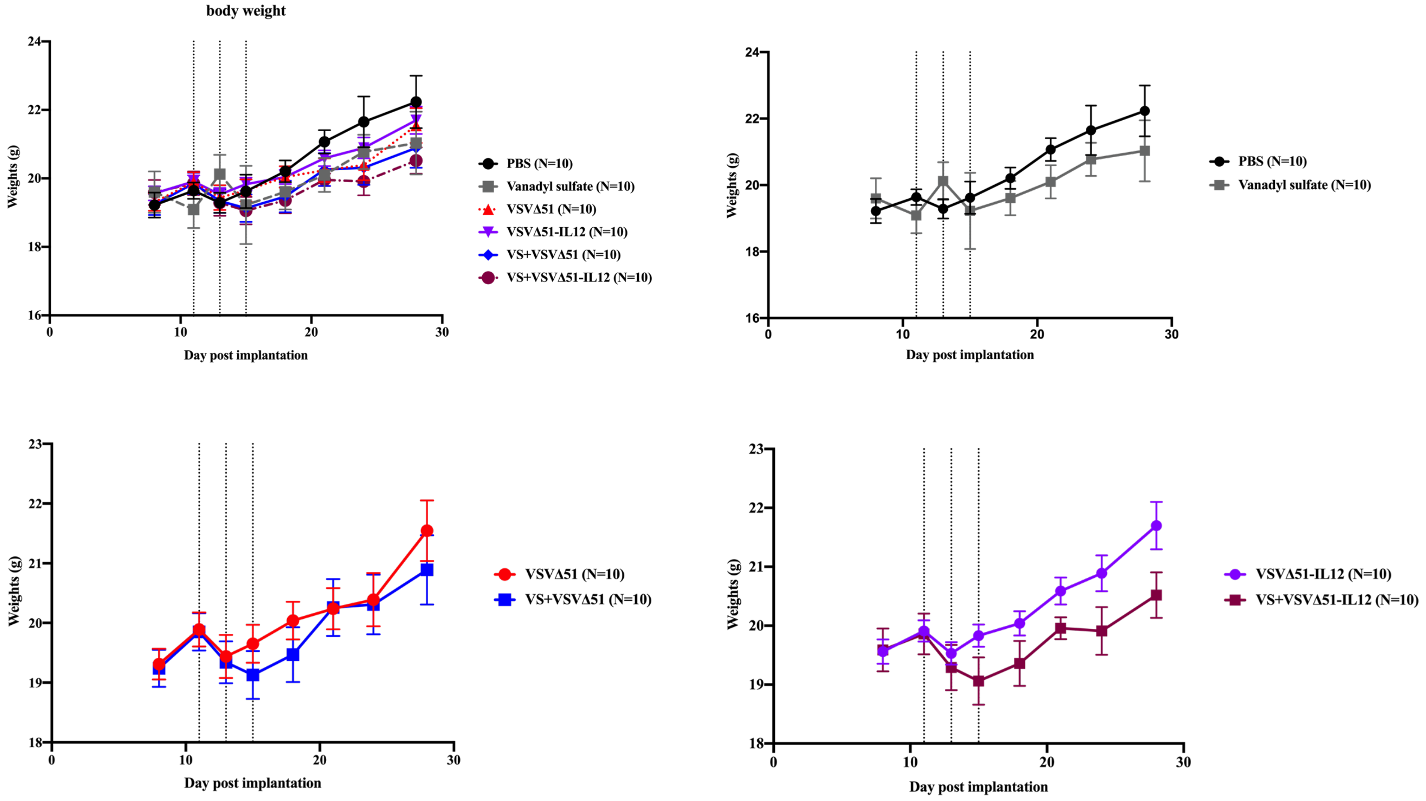

Figure S1: Body weight measurement following treatment with VS/VSVΔ51 or VS/VSVΔ51-IL12 treatment

CT26WT-tumor bearing mice received a total of 3 doses delivered intratumorally of vanadyl sulfate (50mg/kg) and VSVΔ51-fluc (1E8 PFU) or monotreatment injections over 5 days, lines indicate days of the treatment. Body weight was measured three times a week over the first three weeks of the treatment. Graphs show the average of body weight of each group, Mean±SEM; n=10 per group.

cytokines/ ckemokines	day post treatment	PBS vs. VS	PBS vs. VSVΔ51	PBS vs. VS+VSVΔ51	VS vs. VSVΔ51	VS vs. VS+VSVΔ51	VSVd51 vs. VS+VSVΔ51
IFN-γ	1	0.7394	0.1478	<0.0001	0.0111	<0.0001	0.011
	5	0.8883	0.0159	0.0081	0.0017	0.0008	0.9962
	10	0.9311	0.8872	0.2183	0.5482	0.5318	0.046
IL-6	1	0.0034	<0.0001	<0.0001	0.0157	0.0009	0.8159
	5	0.3191	0.3578	<0.0001	0.9999	0.0002	0.0002
	10	0.999	0.0807	0.0073	0.1113	0.0112	0.7809
CXCL10	1	0.03	<0.0001	<0.0001	<0.0001	<0.0001	0.9455
	5	0.69	0.7352	0.0106	0.9998	0.1597	0.1373
	10	0.0006	0.1538	0.0012	0.232	0.9998	0.2888
CXCL9	1	0.4834	<0.0001	<0.0001	<0.0001	<0.0001	0.1075
	5	0.0068	0.0004	0.0114	0.8439	0.9998	0.8141
	10	0.4801	0.961	0.6046	0.7807	0.9985	0.8724
CCL3	1	0.5511	0.0594	0.6391	0.0014	0.0757	0.5705
	5	0.0029	0.827	0.5339	0.0001	<0.0001	0.9552
	10	0.013	0.727	<0.0001	0.1721	0.0019	<0.0001
CCL4	1	0.9833	<0.0001	<0.0001	0.0038	0.0016	0.2138
	5	0.0327	0.9691	0.9658	0.0263	0.2231	0.9083
	10	0.4891	0.9997	0.4514	0.1391	0.0297	0.3638
IL-7	1	0.8818	0.599	0.751	0.8366	0.9728	0.9651
	5	0.093	<0.0001	<0.0001	0.0737	0.0094	0.8767
	10	0.0739	0.0306	0.0139	0.9039	0.882	>0.9999
IL-15	1	0.337	0.973	0.3487	0.1551	>0.9999	0.1658
	5	0.9986	0.2259	0.3963	0.2161	0.4046	0.9875
	10	0.9978	0.5626	0.9686	0.6348	0.9913	0.8224
IL-2	1	0.3954	0.2134	0.5426	0.9885	0.9953	0.9426
	5	0.2294	0.8759	0.0079	0.653	0.4987	0.0604
	10	0.0131	0.98	0.0157	0.0376	>0.9999	0.0442

Figure S2: p value for significant comparison between each condition for each tested cytokines and chemokines following treatment with VS/ VSVΔ51.

Data were analyzed using two-way ANOVA test, * P=0.01, ** P=0.003, *** P=0.001,****P<0.0001

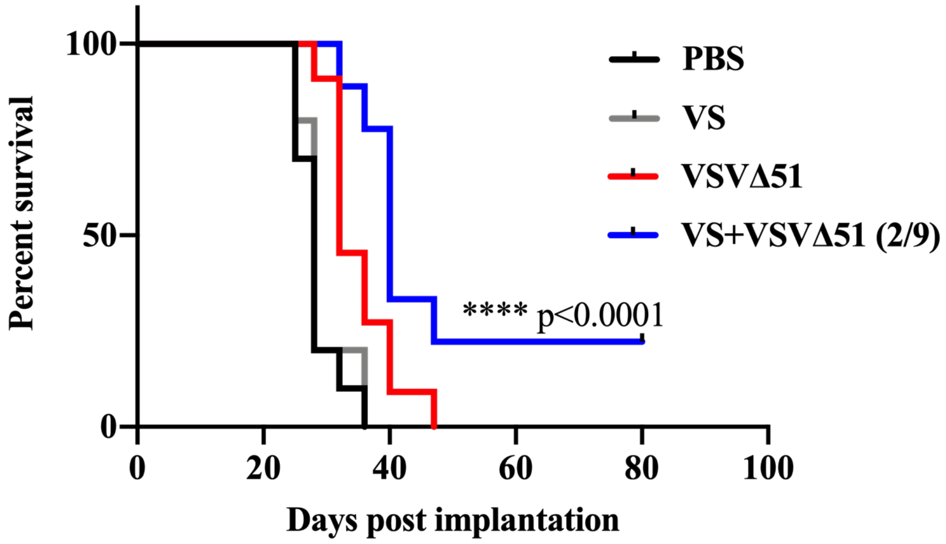

Figure S3: survival rate following treatment with VS/VSVΔ51 treatment
CT26WT-tumor bearing mice received a total of 3 doses delivered intratumorally of vanadyl sulfate (50mg/kg) and VSVΔ51-fluc (1E8 PFU) or monotreatment injections over 5 days. Survival was monitored over time, the percentage of survival was included for each condition, Long rank (Mantel-Cox) test indicated the significant, P<0.0001

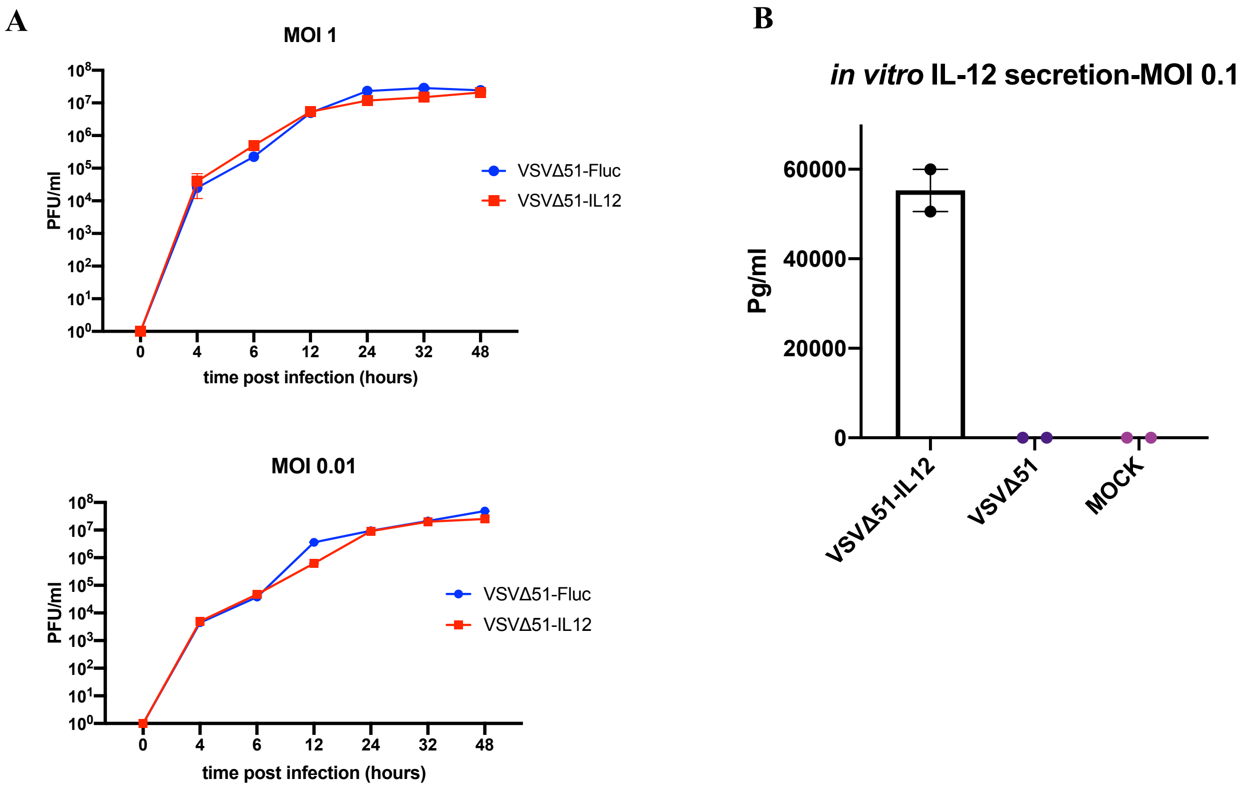

Figure S4: Replication kinetic of VSVΔ51 encoding IL12 and the secretion of IL12 *in vitro.*
(**A**) Replication kinetic of VSVΔ51 encoding fluc or IL12 was performed in multistep or single-step viral growth curves in CT26WT cells. Cells were incubated up to 48 hour post viral infection, 200 µl of supernatant was collected and viral titer in collected supernatant was quantified by high-throughput tittering using a standard plaque assay. Graphs show the plaque performing unit per ml (PFU/ml). (**B**) CT26WT cells were infected with VSVΔ51 encoding fluc or IL12 at MOI 0.1. 24 hrs post-infection, supernatants were collected and the secretion of IL12 was measured by ELISA. Graph shows the pg/ml. Mean±SEM; n=2 per condition.

cytokines/ ckemokines	day post treatment	PBS vs. VS	PBS vs. VSVΔ51 -IL12	PBS vs. VS+VSVΔ51-IL12	VS vs. VSVΔ51-IL12	VS vs. VS+VSVΔ51-IL12	VSVd51 vs. VS+VSVΔ51-IL12
IFN-γ	1	0.6549	<0.0001	<0.0001	<0.0001	<0.0001	0.703
	5	0.8446	<0.0001	<0.0001	<0.0001	<0.0001	0.435
	10	0.9026	0.6053	0.0128	0.946	0.0758	0.2328
IL-6	1	0.0007	<0.0001	<0.0001	0.0046	0.0003	0.8425
	5	0.216	0.2381	<0.0001	>0.9999	<0.0001	<0.0001
	10	0.9985	0.8604	0.9996	0.9236	>0.9999	0.9025
CXCL10	1	0.0216	<0.0001	<0.0001	<0.0001	<0.0001	0.088
	5	0.6616	0.0213	0.0012	0.0005	<0.0001	0.8002
	10	0.0003	0.8973	0.6057	0.0038	0.0197	0.9499
CXCL9	1	0.5808	<0.0001	<0.0001	<0.0001	<0.0001	0.9991
	5	0.0197	<0.0001	<0.0001	0.0602	0.0013	0.5744
	10	0.5777	0.473	0.9979	0.0369	0.4648	0.5862
CCL3	1	0.501	0.9878	0.3473	0.3179	0.0163	0.5417
	5	0.0014	0.9424	0.7005	0.0087	<0.0001	0.3566
	10	0.0073	0.1405	0.0037	0.6737	0.9963	0.5365
CCL4	1	0.9864	0.0003	0.0004	0.0011	0.0015	0.9998
	5	0.0157	0.8066	0.4117	0.1473	0.4494	0.9142
	10	0.3331	0.9732	0.0012	0.1528	<0.0001	0.0049
IL-7	1	0.8503	0.8842	0.4248	0.999	0.6323	0.523
	5	0.0535	<0.0001	0.0071	0.086	0.8885	0.3462
	10	0.0405	0.0295	0.0254	0.9993	0.9977	>0.9999
IL-15	1	0.4559	0.8054	0.3253	0.9389	0.9956	0.8505
	5	0.9991	0.6589	0.8181	0.6792	0.8481	0.9901
	10	0.9985	0.9975	0.9453	>0.9999	0.9753	0.9805
IL-2	1	0.6319	0.9863	0.0002	0.8394	0.0129	0.0008
	5	0.041	0.8403	0.0002	0.2422	0.4336	0.0039
	10	0.9644	0.1281	0.0055	0.3126	0.022	0.606

Figure S5: p value for significant comparison between each condition for each tested cytokines and chemokines following treatment with VS/ VSVΔ51 expressing IL-12.

Data were analyzed using two-way ANOVA test, * P=0.01, ** P=0.003, *** P=0.001,****P<0.0001
